# Human Bone Marrow Mesenchymal Stem/Stromal Cells Exposed to an Inflammatory Environment Increase the Expression of ICAM-1 and Release Microvesicles Enriched in This Adhesive Molecule: Analysis of the Participation of TNF-*α* and IFN-*γ*

**DOI:** 10.1155/2020/8839625

**Published:** 2020-11-30

**Authors:** Juan J. Montesinos, Lucero López-García, Víctor A. Cortés-Morales, Lourdes Arriaga-Pizano, Ricardo Valle-Ríos, Guadalupe R. Fajardo-Orduña, Marta E. Castro-Manrreza

**Affiliations:** ^1^Mesenchymal Stem Cells Laboratory, Oncology Research Unit, Oncology Hospital, National Medical Center (IMSS), Mexico City C.P. 06720, Mexico; ^2^Immunology and Stem Cells Laboratory, FES Zaragoza, National Autonomous University of Mexico (UNAM), Mexico City C.P. 09230, Mexico; ^3^Medical Research Unit in Immunochemistry, Specialty Hospital, National Medical Center (IMSS), Mexico City C.P. 06720, Mexico; ^4^Peripheral Unit for Research in Cancer and Immunology, Research Division, Faculty of Medicine, UNAM, Mexico City C.P. 04360, Mexico; ^5^Immunology and Proteomics Research Laboratory, Hospital Infantil de México Federico Gómez, Mexico City C.P. 06720, Mexico

## Abstract

Bone marrow mesenchymal stem/stromal cells (BM-MSCs) have immunoregulatory capacity; therefore, they have been used in different clinical protocols in which it is necessary to decrease the immune response. This capacity is mainly regulated by TNF-*α* and IFN-*γ*, and it has been observed that cell-cell contact, mainly mediated by ICAM-1, is important for MSCs to carry out efficient immunoregulation. Therefore, in the present work, we analyzed the effect of TNF-*α* alone or in combination with IFN-*γ* on the expression of ICAM-1. Besides, given the importance of cell contact in the immunoregulatory function of MSCs, we analyzed whether these cells release ICAM-1^+^ microvesicles (MVs). Our results show for the first time that TNF-*α* is capable of increasing the early expression of ICAM-1 in human BM-MSCs. Also, we observed that TNF-*α* and IFN-*γ* have a synergistic effect on the increase in the expression of ICAM-1. Furthermore, we found that BM-MSCs exposed to an inflammatory environment release MVs enriched in ICAM-1 (MVs-ICAM-1^high^). The knowledge generated in this study will contribute to the improvement of *in vitro* conditioning protocols that favor the therapeutic effect of these cells or their products.

## 1. Introduction

Bone marrow (BM) mesenchymal stem/stromal cells (MSCs) have immunoregulatory capacity, and due to this property, they have been used in various preclinical models and clinical trials [1, 2] in which decreasing the immune response is required, to avoid tissue damage and stimulate their regeneration. *In vitro* and *in vivo* studies have shown that BM-derived MSCs (BM-MSCs) and other tissues are capable of modulating the function of immune system cells, including neutrophils, natural killer cells, monocytes, macrophages, dendritic cells (DC), and T and B lymphocytes, resulting in the generation of an anti-inflammatory environment [3–5].

The immunoregulatory function of BM-MSCs is carried out through independent or dependent mechanisms of cell-cell contact. Molecules such as the intracellular enzyme indoleamine-2-3-dioxygenase (IDO), prostaglandin (PGE2), transforming growth factor-beta (TGF-*β*), hepatocyte growth factor (HGF), human leukocyte antigen-G5 (HLA-G5), interleukins IL-6 and IL-10, and galectins participate in the former. On the other hand, the mechanisms that require cell contact are mediated by membrane molecules such as programmed death ligand-1 (PDL-1), human leukocyte antigen-G1 (HLA-G1), CD73, Jagged-1, vascular cell adhesion molecule-1 (VCAM-1), and intercellular adhesion molecule-1 (ICAM-1/CD54) [6–8]. In particular, it has been shown that ICAM-1 increases migration and strengthens the adhesion of MSCs to immune cells, and its inhibition affects immunoregulation [9–11]. Currently, it is accepted that cell contact is essential for MSCs to carry out optimal immunoregulatory effects [12, 13]. Therefore, in addition to the above mechanisms, it has been proposed that these cells release extracellular vesicles (EVs). EVs include exosomes, microvesicles (MVs), and apoptotic bodies. It has been shown that exosomes and MVs are capable of transporting biomolecules, establishing contact with target cells and influencing their biological behavior, which is why they are considered an important mechanism of paracrine and distant cellular communication. In this regard, the presence of immunoregulatory molecules such as IDO and TGF-*β* has been reported in EVs released by MSCs [14–16].

It has been observed that the immunoregulatory capacity of MSCs is triggered by the inflammatory environment, mainly by the presence of cytokines such as IFN-*γ*, TNF-*α*, IL-1, and IL-17, which promote the transition of resting MSCs to an activated state with immunoregulatory capacity [17–22]. Therefore, previous studies have examined the effect of these cytokines in *in vitro* conditioning protocols to induce and increase the immunoregulatory capacity of MSCs to promote the therapeutic effect of these cells. Most studies have focused on analyzing the effect of IFN-*γ* [3, 17, 21, 23]. However, various observations suggest that exposure to this cytokine is not sufficient for MSCs to be properly activated and achieve their maximum immunoregulatory potential; therefore, there is a need for concomitant stimulation with TNF-*α* [24–27]. TNF-*α* is one of the first cytokines secreted by immune system cells during inflammation and can increase (prime) or decrease (desensitize or tolerate) the ability of cells to respond to other environmental stimuli [28–30]. It has been shown that this cytokine induces the expression of adhesion molecules such as ICAM-1 in the vascular endothelium and promotes the recruitment of lymphocytes to sites of inflammation. The participation of TNF-*α* in the induction and resolution of inflammation is important in the maintenance of homeostasis because an excess of this cytokine has been associated with the pathogenesis of inflammatory and autoimmune diseases [29].

Despite the importance of TNF-*α* as an inflammatory cytokine capable of regulating the response of cells to other stimuli, few studies have analyzed the direct effect of this cytokine on MSC functions. In this regard, it has been reported that the stimulation of MSCs derived from human BM or adipose tissue with TNF-*α* induces an increase in the expression of growth factors such as VEGF, HGF, and IGF-1 [31], which increases the regeneration potential of MSCs [32]. Besides, it induces an increase in the secretion of TGF*β* and IL-10 in rat umbilical cord MSCs [33]. In the BM-MSCs of rats, TNF-*α* facilitates the migration capacity and increases the expression of ICAM-1 and VCAM-1 [9]. It has even been argued that this cytokine provides the initial stimulus in the priming of MSCs [34]. Therefore, the present work analyzed the effect of TNF-*α* alone or in combination with IFN-*γ* on the expression of ICAM-1, an important molecule in the immunoregulation of MSCs. Furthermore, given the importance of cell contact in MSC functions, we analyzed whether ICAM-1 is enriched in the MVs released by MSCs exposed to an inflammatory environment. Because none of these aspects have been analyzed in human BM-MSCs, our study contributes to the knowledge of the influence of the microenvironment on the functions of BM-MSCs, which will allow improving *in vitro* conditioning strategies to produce cells or cellular products capable of functioning in different therapeutic scenarios.

## 2. Materials and Methods

### 2.1. Isolation and Culture of BM-MSCs

BM samples were obtained from 3 volunteer donors according to the ethical guidelines of Villa Coapa Hospital, Mexican Social Security Institute (IMSS). The project was approved by the ethics and biosafety commission of the FES Zaragoza, UNAM (FESZ/DEPI/CI/280/17, December 8, 2017). Mononuclear cells (MNC) were isolated from BM as previously described [35], after which the cells were resuspended in HyClone Dulbecco's Modified Eagle Medium (DMEM) with low glucose (GE Healthcare Life Sciences) containing 10% fetal bovine serum (FBS; Gibco BRL), 4 mM L-glutamine, 100 U/mL penicillin, 100 mg/mL streptomycin, and 100 mg/mL gentamicin (all reagents were obtained from Gibco BRL); the cells were seeded at a density of 2.0 × 10^5^ cells/cm^2^ in T-75 culture flasks (Corning, Inc./Costar; New York, NY) and incubated at 37°C with 5% CO_2_. After 4 days of culture, the nonadherent cells were removed, and fresh medium was added to the cultures. Once the cultures reached 80-85% confluence, the cells were harvested (0.05% trypsin, 0.53 mM EDTA; Gibco BRL) and subcultured at a density of 2000 cells/cm^2^ in a 100 mm Petri dish (Corning); fresh medium was added every four days. At the second passage, the cells were harvested, analyzed, and cryopreserved for future use. The BM-MSCs of passages 3 and 4 were used for the experiments.

### 2.2. Characterization of BM-MSCs

Immunophenotypic characterization and differentiation capacities of MSCs was performed according to the methodology described by Montesinos et al. [35]. Monoclonal antibodies conjugated to FITC, PE, or APC against surface markers characteristic of MSCs (CD90, CD105, CD73, CD13, HLA-I, HLA-II, CD45, CD31, CD34, CD14, and CD54/ICAM-1 (BD Biosciences, San Diego, CA)) were used for immunophenotypic characterizations, as described in Flow Cytometry Analysis of Cells.

Adipogenic and osteogenic differentiation was induced with Stem Cell Kits (Stemcell Technologies, Inc., Vancouver, BC, Canada). Adipogenic differentiation was determined by visualizing the presence of Oil Red O-stained lipid vacuoles (Sigma-Aldrich, St. Louis, MO). Osteogenic differentiation was determined by alkaline phosphatase activity which was detected using SIGMAFAST™ BCIP/NBT (Sigma-Aldrich). Chondrogenic differentiation was induced with a commercial induction medium supplemented with 10 ng/mL of TGF-*β* (both reagents of Cambrex Bio Science). The resulting micromasses were fixed, dehydrated, embedded in paraffin, and sliced. Cross sections of 5 *μ*m were stained with Alcian blue dye (Sigma-Aldrich).

### 2.3. Collection and Stimulation of Peripheral Blood Mononuclear Cells (PBMCs)

PBMCs were obtained from the peripheral blood samples of three volunteer donors by density gradient with Ficoll-Paque Plus (specific gravity < 1.077 g/mL; GE Healthcare Bio-Sciences AB, Uppsala, Sweden). PBMCs were kept in RPMI medium (RPMI 1640, 8% fetal calf serum, 2 mM L-glutamine, 100 U/mL of penicillin, 100 mg/mL of streptomycin, and 100 mg/mL of gentamicin (all reagents were obtained from Gibco BRL)) for 24 hours. PBMCs (4 × 10^5^ cells) were activated with 5 *μ*g/mL phytohemagglutinin (PHA), and supernatants were collected at 24, 48, and 96 hours of activation and placed at -80°C. These samples were used to determine the concentration of cytokines.

### 2.4. Quantitative Analysis of TNF-*α* and IFN-*γ*

Cytokine analysis was performed using a Cytometric Bead Array kit (BD Biosciences) according to the supplier's instructions. The samples were analyzed on a FACSCanto II Flow Cytometer (BD Biosciences) and analyzed with LEGENDplex v7.1 software (BioLegend, USA).

### 2.5. *In Vitro* Stimulation of MSCs

BM-MSCs were seeded in 24-well plates at a density of 2000 cells/cm^2^. When the cultures reached 90% confluence, the monolayer was washed with PBS, and fresh medium supplemented with 10% FBS was added. These cultures were maintained under one of the following stimulation conditions: (a) 5, 10, 20, or 90 ng/mL IFN-*γ* for 24, 48, and 72 hours; (b) 0.5 or 1.0 ng/mL TNF-*α* for 3, 6, 12, 24, 48, and 72 hours; or (c) 5 ng/mL and 90 ng/mL IFN-*γ* alone or in combination with 0.5 or 1.0 ng/mL TNF-*α* for 24, 48, and 72 hours. BM-MSC cultures which were maintained only with medium for 24, 48, and 72 hours were used as a control. The cells were harvested and processed to analyze the expression of HLA-I and ICAM-1 by flow cytometry.

BM-MSCs were seeded in Petri dishes (p100) at a density of 2000 cells/cm^2^. When the cultures reached 90% confluence, the monolayer was washed with PBS, and fresh medium supplemented with 10% FBS, previously filtered through a 0.2 *μ*m membrane, was added. Control cells or BM-MSCs activated with 10 ng/mL IFN-*γ* were maintained in culture for 72 hours. The supernatants of these cultures were used to isolate MVs.

### 2.6. Flow Cytometry Analysis of Cells

To analyze the immunophenotype of BM-MSCs, as well as changes in HLA-I and ICAM-1 expression in cytokine-stimulated BM-MSCs, extracellular staining was performed. A total of 1.0 × 10^5^ MSCs were resuspended in 100 *μ*L of phosphate-buffered saline with 3% FBS and 1 mM EDTA (cytometry buffer) and incubated for 20-30 min with the appropriate antibodies. Next, the cells were washed with 1 mL of cytometry buffer and fixed with FACS Lysing Solution (BD Biosciences). The cells were subsequently washed with 1 mL of cytometry buffer. The samples were analyzed using a BD Bioscience FACSCanto II Flow Cytometer (BD Biosciences), and at least 10,000 events were collected. The data were analyzed with FlowJo 7.6.1 software, and the percentages of positive cells and mean fluorescence intensity (MFI) were obtained.

### 2.7. Isolation and Characterization of Microvesicles

MVs were obtained according to the methodology proposed by Wang et al. [36] from supernatants of unstimulated BM-MSCs or activated with 10 ng/mL IFN-*γ*. The supernatants were collected and centrifuged consecutively at 500 × *g* for 15 minutes, at 2000 × *g* for 20 minutes, and at 17,000 × *g* for 60 minutes; the pellets resulting from the last centrifugation were washed and resuspended in PBS for further characterization.

The characterization of the MVs was performed by flow cytometry using the violet side scatter (Violet-SSC) configuration on a CytoFLEX XL cytometer (Beckman Coulter). The equipment was configured to detect fluorescent nanoparticles, and its resolution capacity was verified by using 130 nm, 220 nm, 450 nm, 880 nm, and 1330 nm fluorescent nanospheres (yellow, flow cytometry grade) (Spherotech). To reduce the background, before starting the acquisition of MVs, the cytometer was washed with filtered (0.2 *μ*m membrane) sheath fluid for 10 minutes. Likewise, a 2-minute wash was performed between each acquired sample. The selection of the MV region (MVs-Gate) was determined by the detection of Violet-SSC and the forward scatter component (FSC). To analyze the presence of HLA-I and ICAM-1, MVs were incubated for 30 minutes at 4°C with monoclonal antibodies coupled to FITC and PE, respectively. Subsequently, the MVs were washed with 1 mL of previously filtered PBS and stored in a refrigerator for a maximum of 24 hours. A total of 1 × 10^6^ events were acquired by the CytoFLEX XL cytometer. The data were analyzed with CytExpert 2.0 software, and the percentage of expression and the MFI were obtained.

### 2.8. Statistical Analysis

The data are expressed as the mean and standard error of the mean (SEM). Statistical analyses were performed using GraphPad Prism 5. Comparisons between groups were performed by the paired *t*-test or Mann–Whitney *U* test. A *p* value < 0.05 was considered to be significant.

## 3. Results

### 3.1. Characterization of BM-MSCs

**Figure 1:**
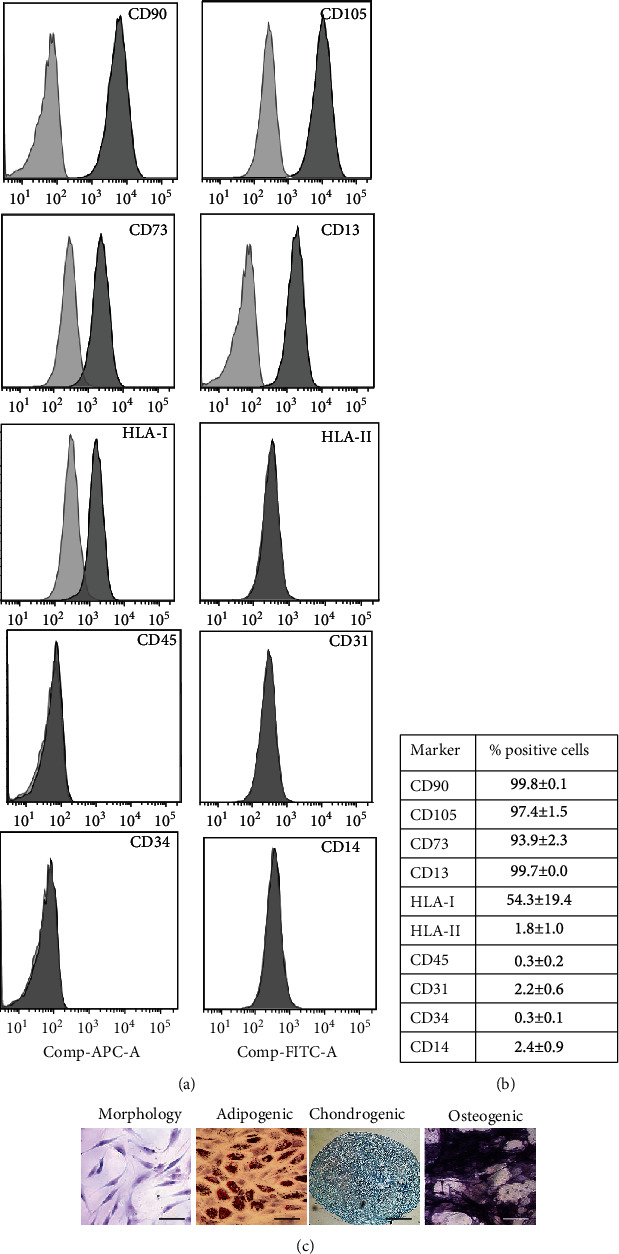
Characterization of BM-MSCs. Analysis of the immunophenotype by flow cytometry: (a) representative histograms of markers analyzed and (b) the mean ± SEM of the expression percentages (*n* = 3). (c) Representative photos of the morphology as well as the adipogenic (scale bar = 100 *μ*m), chondrogenic (scale bar = 200 *μ*m), and osteogenic (scale bar = 100 *μ*m) differentiation capacity are shown.

The MSCs obtained from human BM showed fibroblast morphology, and flow cytometric analysis showed that they expressed the markers CD105, CD90, CD73, and HLA-I and were also negative for HLA-II, CD45, CD31, and CD14 (Figures 1(a) and 1(b)). Besides, they showed adipogenic, osteogenic, and chondrogenic differentiation capacity (Figure 1(c)). In Supplementary Figure 1, the negative controls of the adipogenic and osteogenic differentiation protocols are shown. Our results are consistent with the guidelines established by the International Society for Cellular Therapy [37].

### 3.2. TNF-*α* Is the First Cytokine Secreted by Activated PBMCs

**Figure 2:**
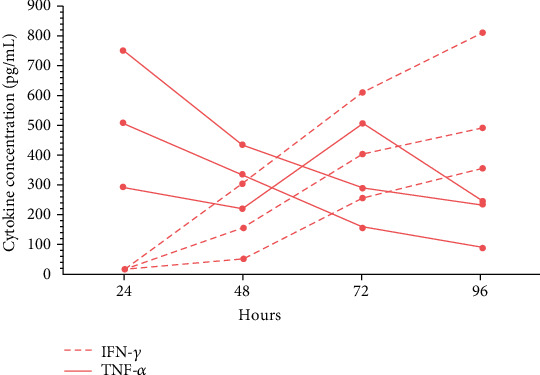
Changes in the secretion of TNF-*α* and IFN-*γ* by activated peripheral blood mononuclear cells. PHA-activated PBMCs (4 × 10^5^) were cultured for 24, 48, 72, and 96 hours. After the activation time, supernatants were obtained to quantify the concentration of TNF-*α* and IFN-*γ* by CBA. The results obtained from three different donors are shown (each line represents a donor) (*n* = 3).

It has been proposed that TNF-*α* and IFN-*γ* released by activated T lymphocytes are the main cytokines involved in the increase in the immunoregulatory capacity of MSCs [27, 34, 38, 39]. Therefore, to determine the most appropriate concentrations of TNF-*α* and IFN-*γ* to be used in this study, we decided to quantify them in the supernatants of PBMCs activated with PHA for 24, 48, 72, and 96 hours. The results showed high concentrations of TNF-*α* at 24 hours of activation; the levels decreased at 48, 72, and 96 hours (Figure 2). However, IFN-*γ* secretion increased after 48 hours, and this trend was maintained over time, with the highest concentration observed at 96 hours (Figure 2). Based on these results and data previously reported by our working group [13] and the literature, the concentrations of TNF-*α* (0.5 and 1 ng/mL) and IFN-*γ* (5, 10, 20, and 90 ng/mL) used in this study were established.

### 3.3. Stimulation with a Low Concentration of IFN-*γ* Is Sufficient to Activate BM-MSCs

**Figure 3:**
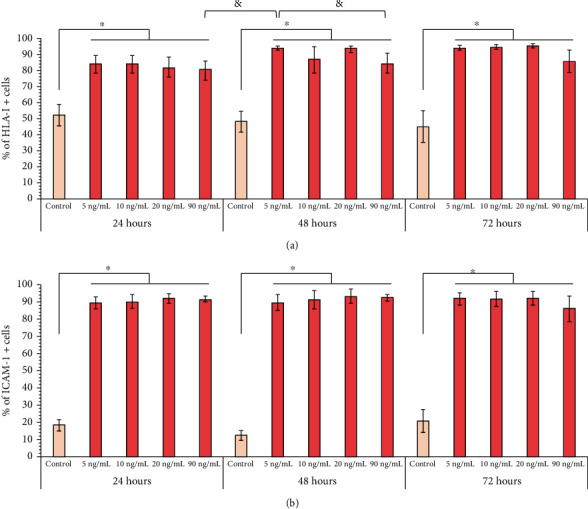
IFN-*γ* stimulation increases the expression of HLA-I and ICAM-1 in BM-MSCs. BM-MSC cultures were treated with 5, 10, 20, and 90 ng/mL IFN-*γ* for 24, 48, and 72 hours. The basal expression of HLA-I and ICAM-1 in BM-MSCs not stimulated and cultured for the same times was used as the control. (a) The mean ± SEM of the percentage of HLA-I^+^ cells. (b) The mean ± SEM of the percentage of ICAM-1^+^ cells. ^∗^*p* < 0.05 with respect to the control; ^&^*p* < 0.05 between 5 ng/mL IFN-*γ* for 48 h and 90 ng/mL IFN-*γ* for 24 and 48 hours. *n* = 3-10 (independent experiments).

Resting MSCs constitutively express low levels of HLA-I [7] and adhesive ligands such as ICAM-1 [40], whose expression increases in MSCs exposed to an inflammatory environment containing IFN-*γ* [13, 41]. Several studies have used the increase in major histocompatibility complex (MHC) expression as a marker of MSC activation [7, 12, 38]. Therefore, we first analyzed the effect of different concentrations of IFN-*γ* (5, 10, 20, and 90 ng/mL) on HLA-I expression to determine whether low concentrations of this cytokine could effectively increase the expression of this molecule in BM-MSCs. We observed that all IFN-*γ* concentrations increased the percentage of HLA-I^+^ cells at 24, 48, and 72 hours of treatment compared to that in the control (*p* < 0.05) (Figure 3(a)). Even stimulation with 5 ng/mL IFN-*γ* for 48 hours induced higher percentage of HLA-I^+^ cells (94.2% ± 1.2%) than those with 90 ng/mL for 24 (79.9% ± 6.1%) and 48 (84.1% ± 6.0%) hours.

Subsequently, we examined whether these concentrations of IFN-*γ* also stimulate the expression of ICAM-1 in BM-MSCs. We observed that a low percentage of BM-MSCs expressed ICAM-1 constitutively and that stimulation with low concentrations of IFN-*γ* induced an increase in the percentage of ICAM-1^+^ cells at 24 (18.2% ± 3.3% vs. 88.8% ± 4.0%), 48 (12.2% ± 2.5% vs. 89.1% ± 5.0%), and 72 (20.5% ± 6.7% vs. 91.2% ± 4.3%) hours; there was no significant difference between the concentrations and times analyzed (Figure 3(b)). Our results indicate that the activation of MSCs, reflected in the increase in the expression of HLA-I and ICAM-1, was achieved with the lowest concentration of IFN-*γ* (5 ng/mL) used in this study, which is closer to the levels found in a physiological context.

### 3.4. TNF-*α* Stimulates the Early Expression of ICAM-1 but Not HLA-I in BM-MSCs

**Figure 4:**
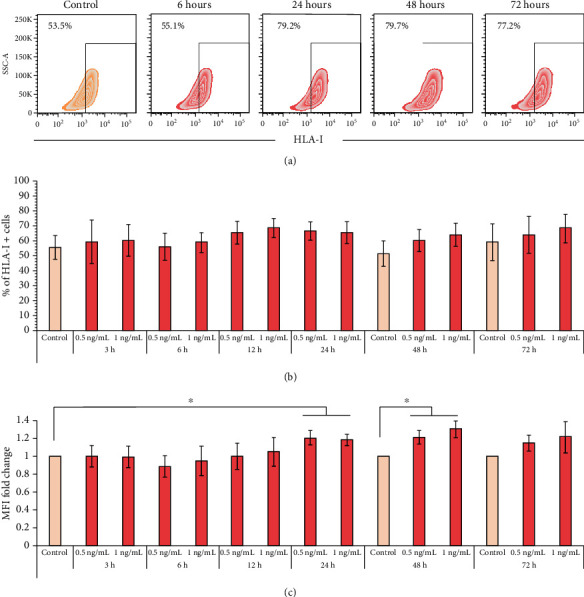
TNF-*α* has a late effect on HLA-1 expression in BM-MSCs. BM-MSCs were stimulated with 0.5 or 1.0 ng/mL TNF-*α* for the indicated times. The basal expression of HLA-I in nonstimulated BM-MSCs cultured for 24, 48, and 72 hours was used as the control. (a) Representative graphics of the changes in the expression of HLA-I in BM-MSCs stimulated with 1.0 ng/mL TNF-*α*. (b) The mean ± SEM of the percentage of HLA-I^+^ cells. (c) The mean ± SEM of the fold change in HLA-I MFI. ^∗^*p* < 0.05 compared to the control. *n* = 3-7 (independent experiments).

TNF-*α* is another cytokine associated with the increased immunoregulatory function of BM-MSCs; therefore, we decided to analyze its effect on the expression of HLA-I and ICAM-1. Our results showed that treatment with 0.5 or 1 ng/mL TNF-*α* did not affect the percentage of HLA-I^+^ cells at the analyzed times (Figures 4(a) and 4(b)). However, although the percentage of HLA-I^+^ cells was not modified relating to the control, we did observe an increase in the level of HLA expression, an event that is reflected in a higher MFI at 24 (0.5 ng/mL: 1.2‐fold ± 0.07; 1.0 ng/mL: 1.1‐fold ± 0.05) and 48 (0.5 ng/mL: 1.1‐fold ± 0.07; 1.0 ng/mL: 1.2‐fold ± 0.1) hours (Figures 4(a) and 4(c)).

**Figure 5:**
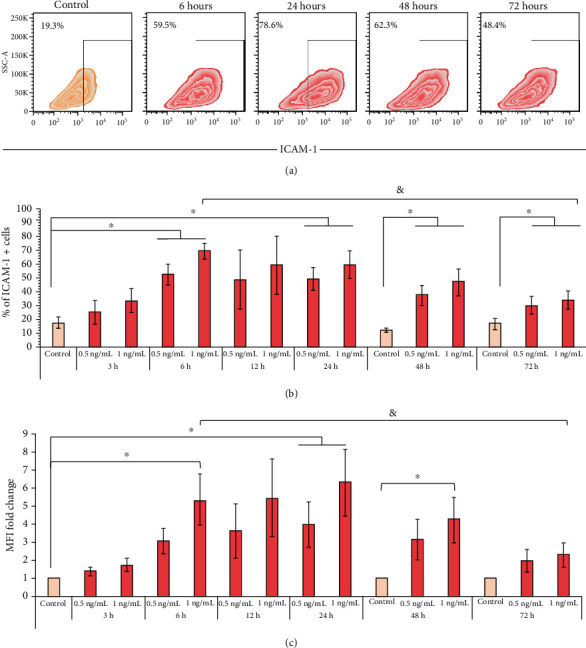
TNF-*α* induces the early expression of ICAM-1 in BM-MSCs. BM-MSCs were stimulated with 0.5 or 1.0 ng/mL TNF-*α* for the indicated times. The basal expression of ICAM-1 in nonstimulated BM-MSCs cultured for 24, 48, and 72 hours was used as the control. (a) Representative graphics of the changes in the expression of ICAM-1 in BM-MSCs stimulated with 1.0 ng/mL TNF-*α*. (b) The mean ± SEM of the percentage of ICAM-1^+^ cells. (c) The mean ± SEM of the fold change in ICAM-1 MFI. ^∗^*p* < 0.05 compared to the control; ^&^*p* < 0.05 between 1 ng/mL TNF-*α* for 6 hours and 1 ng/mL TNF-*α* for 72 hours. *n* = 3-7 (independent experiments).

On the other hand, the stimulation of BM-MSCs with 0.5 or 1.0 ng/mL TNF-*α* induced an increase in the percentage of ICAM-1^+^ cells after 3 hours of treatment. This effect was statistically significant at 6 (control (18.8% ± 3.6%) vs. 0.5 ng/mL (52.0% ± 7.7%) and 1 ng/mL (69.2% ± 5.7%)), 24 (control (18.8% ± 3.6%) vs. 0.5 ng/mL (49.2% ± 8.3%) and 1 ng/mL (59.2% ± 9.9%)), 48 (control (12.4% ± 2.0%) vs. 0.5 ng/mL (37.4% ± 7.1%) and 1 ng/mL (46.9% ± 9.6%)), and 72 (control (16.8% ± 3.7%) vs. 0.5 ng/mL (29.9% ± 6.6%) and 1 ng/mL (34.0% ± 6.8%)) hours (Figure 5(b)). Besides, we observed an increase in the level of ICAM-1 expression in BM-MSCs. Stimulation with 0.5 ng/mL TNF-*α* induced a significant increase in ICAM-1 expression only at 24 hours (3.9‐fold ± 1.2), whereas the stimulation with 1 ng/mL TNF-*α* resulted in a statistically significant increase in ICAM-1 expression at 6 (5.3‐fold ± 1.4), 24 (6.3‐fold ± 1.8), and 48 (4.2‐fold ± 1.2) hours (Figure 5(c)). However, it is interesting to note that stimulation with 1 ng/mL TNF-*α* at 72 hours induced a smaller increase in the percentage of ICAM-1^+^ cells (34.0% ± 6.8%) compared to that observed at 6 hours (69.2% ± 5.4%). Even the expression levels of ICAM-1 detected at 72 hours were no longer statistically significant relating to the control (2.2‐fold ± 0.7) and were lower than those observed at 6 hours of stimulation with the same concentration of TNF-*α* (5.3‐fold ± 1.4) (Figures 5(b) and 5(c)). These data indicate that TNF-*α* induces the early expression of ICAM-1 in BM-MSCs, while the effect on HLA-I is late.

### 3.5. TNF-*α* and IFN-*γ* Exert a Synergistic Effect on the Induction of ICAM-1 Expression in BM-MSCs

**Figure 6:**
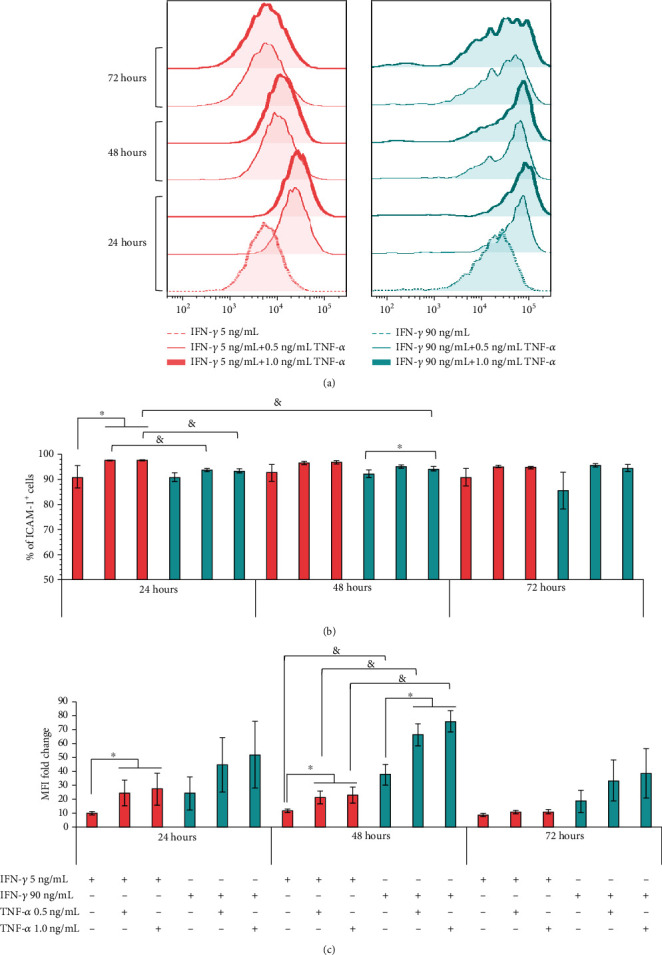
TNF-*α* and IFN-*γ* exert a synergistic effect on the induction of ICAM-1 expression in BM-MSCs. BM-MSCs were stimulated with 5 and 90 ng/mL IFN-*γ* alone (control) or in combination with 0.5 and 1.0 ng/mL TNF-*α* for 24, 48, and 72 hours. (a) Representative histograms of the changes in the expression of ICAM-1 in stimulated BM-MSCs with IFN-*γ* alone or in combination with TNF-*α* at the concentrations and times indicated. (b) The mean ± SEM of the percentage of ICAM-1^+^ cells; ^∗^*p* < 0.05 with respect to the control; ^&^*p* < 0.05 between 5 ng/mL IFN-*γ* plus 0.5 or 1 ng/mL TNF-*α* and 90 ng/mL IFN-*γ* plus 0.5 or 1 ng/mL TNF-*α* for 24 hours and between 5 ng/mL IFN-*γ* plus 1 ng/mL TNF-*α* 24 hours and 90 ng/mL IFN-*γ* plus 1 ng/mL TNF-*α* for 48 hours. (c) The mean ± SEM of the fold change in ICAM-1 MFI. ^∗^*p* < 0.05 with respect to the control. ^&^*p* < 0.05 between 5 ng/mL IFN-*γ* and MFI-90 ng/mL IFN-*γ* for 48 hours and between 5 ng/mL IFN-*γ* plus 0.5 or 1 ng/mL TNF-*α* and 90 ng/mL IFN-*γ* plus 0.5 or 1 ng/mL TNF-*α* for 48 hours. *n* = 3-7 (independent experiments).

It has been proposed that ICAM-1 increases migration and strengthens the adhesion of MSCs to immune cells, favoring their immunoregulatory potential [9, 10, 42, 43]. Therefore, we evaluated whether TNF-*α* and IFN-*γ* exerted a synergistic effect on the expression of ICAM-1 in BM-MSCs. For this, we used 5 ng/mL (low) and 90 ng/mL (high) IFN-*γ*, alone or in combination with 0.5 or 1.0 ng/mL TNF-*α*, for 24, 48, and 72 hours. We observed that BM-MSCs treated with 5 or 90 ng/mL IFN-*γ* increased, at the same level, the percentage of ICAM-1^+^ cells (Figure 6(a)). However, treatment for 48 hours with 90 ng/mL IFN-*γ* induces a significantly greater increase in the expression of ICAM-1 (37.6‐fold ± 7.3) than that observed using low concentrations of this cytokine (11.2‐fold ± 1.5), while at 24 (9.7‐fold ± 1.5 vs. 24.0‐fold ± 11.7; 5 and 90 ng/mL, respectively) and 72 (8.3‐fold ± 1.0 vs. 18.2‐fold ± 8.0; 5 and 90 ng/mL, respectively) hours, no significant differences were identified in the level of ICAM-1 expression (Figure 6(b)).

Interestingly, by stimulating cells with both cytokines, greater induction of ICAM-1 was achieved. The analysis of the percentage of ICAM-1^+^ cells (Figure 6(b)) shows a synergistic effect in BM-MSCs stimulated for 24 hours with 0.5 or 1.0 ng/mL TNF-*α* plus 5 ng/mL IFN-*γ* (97.6% ± 0.14% and 97.7% ± 0.17%, respectively), compared to those stimulated only with IFN-*γ* (91.0% ± 4.3%). Besides, at 48 and 72 hours, the same trend was still observed, but without being statistically significant, while the use of 0.5 or 1.0 ng/mL TNF-*α* plus 90 ng/mL IFN-*γ* (93.8% ± 0.4% and 93.4% ± 0.9%, respectively) did not induce a synergistic effect on the percentage of ICAM-1^+^ cells at 24 hours of treatment, compared to those stimulated only with IFN-*γ* (90.0% ± 1.6); even the increase was less than that recorded with 5 ng/mL IFN-*γ* plus 0.5 or 1.0 ng/mL TNF-*α* (97.6% ± 0.14% and 97.7% ± 0.17%, respectively). However, when using high concentrations of IFN-*γ* plus 1.0 ng/mL TNF-*α* for 48 hours, a statistically significant synergistic effect was observed (92.1% ± 1.6% vs. 94.3% ± 0.9%), although the increase in the percentage of ICAM-1^+^ cells was still lower than that recorded at 24 hours of stimulation with 1 ng/mL TNF-*α* in combination with low concentrations of IFN-*γ* (97.7% ± 0.1%). It is important to mention that the stimulation of BM-MSCs, for 24 and 48 hours with both cytokines, does not exert a synergistic effect on the percentage of HLA-I^+^ cells (Supplementary Figure 2a).

The analysis of the MFI of ICAM-1 indicates a significant synergistic effect in the expression level of this immunoadhesive molecule in BM-MSCs stimulated with low concentrations of IFN-*γ* plus 0.5 or 1.0 ng/mL TNF-*α* for 24 (24.0‐fold ± 9.1 and 26.7‐fold ± 11.4, respectively) and 48 (20.0‐fold ± 4.6 and 22.7‐fold ± 5.8, respectively) hours, compared to those stimulated only with 5 ng/mL IFN-*γ* for 24 (9.7‐fold ± 1.5) and 48 (11.2‐fold ± 1.5) hours (Figure 6(c)). On the other hand, in BM-MSC (Figure 6(c)) stimulated with high concentrations of IFN-*γ* plus 0.5 or 1.0 ng/mL of TNF-*α*, a synergistic effect is observed at all times analyzed, although it is statistically significant only at 48 hours of treatment (37.6‐fold ± 7.3 vs. 66.0‐fold ± 8.0 and 75.5‐fold ± 7.9). Likewise, we observed that the increase in the expression level of ICAM-1 in cells stimulated with 90 ng/mL IFN-*γ* alone or in combination with 0.5 or 1.0 ng/mL TNF-*α* is greater (*p* < 0.05) than that induced with low concentrations of this cytokine alone or in combination with 0.5 or 1.0 ng/mL TNF-*α* (Figure 6(c)). However, it is important to mention that the same effect is observed in the expression level of HLA-I, in BM-MSC stimulated for 48 hours with high concentrations of IFN-*γ* alone or in combination with 0.5 or 1.0 ng/mL TNF-*α* (Supplementary Figure 2b). Together, our results indicate the importance of TNF-*α* in increasing the adhesive capacity of BM-MSCs by exerting a synergistic effect with IFN-*γ* and inducing greater expression of ICAM-1.

### 3.6. BM-MSCs Exposed to an Inflammatory Environment Release MVs Enriched in ICAM-1

**Figure 7:**
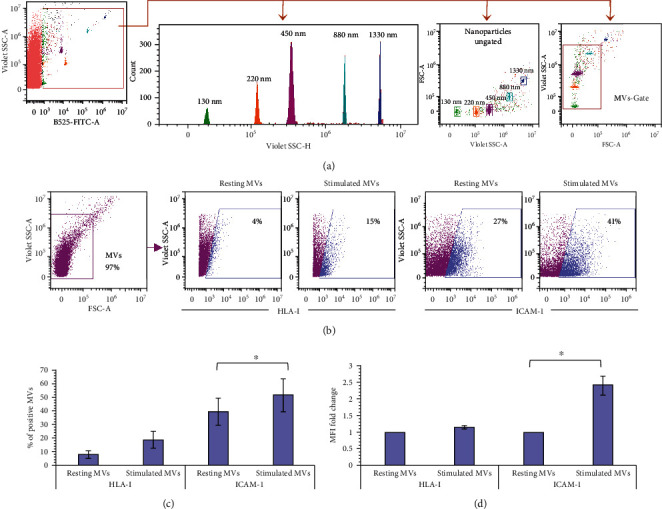
BM-MSCs exposed to an inflammatory environment release MVs enriched in ICAM-1. MVs were obtained from unstimulated BM-MSC supernatants (resting MVs) or stimulated with IFN-*γ* (stimulated MVs) for 72 hours. Characterization of the MVs by flow cytometry: (a) a dot plot representative of the ability of the cytometer to discriminate the fluorescent nanoparticles from noise is shown. From the region corresponding to the nanoparticles, a histogram and a dot plot representative of the resolution capacity of the different sizes of nanoparticles are obtained. Likewise, the graph showing the region corresponding to 130-1000 nm MVs (MVs-Gate) is displayed. (b) A representative dot plot of the MVs-Gate (pink) is shown. From this region, the fluorescence channels of interest are displayed. The MVs-HLA-I^+^ and MVs-ICAM-1^+^ cells obtained from resting or stimulated BM-MSC supernatants are shown in blue. (c) The mean ± SEM of the percentage of MVs-HLA-I^+^ and MVs-ICAM-1^+^. (d) The mean ± SEM of the fold change in HLA-I MFI and ICAM-1 MFI in MVs. ^∗^*p* < 0.05, *n* = 3 (independent experiments).

The participation of exosomes and MVs has been proposed as a mechanism of cellular communication between MSCs and immune cells, which favors efficient immunoregulation, even at distant sites. Therefore, we analyzed whether BM-MSCs are capable of releasing MVs with ICAM-1 on their surface (MV-ICAM-1^+^), which could facilitate the interaction between MSCs and their target cells. Through flow cytometry, we determined that the MVs obtained from unstimulated BM-MSC supernatants (resting MVs) or stimulated with IFN-*γ* (stimulated MVs) had a size of 130-1000 nm (Figure 7(a)). Furthermore, in the stimulated BM-MSC supernatants, there was an increasing trend for the percentages of HLA-I^+^ MVs (7.9% ± 2.8% vs. 18.8% ± 6.2%) and ICAM-1^+^ MVs (39.4% ± 10.4 vs. 51.1% ± 12.1), but this increase is only statistically significant for ICAM-1 (Figures 7(b)–7(c)). Also, the MFI analysis indicated that stimulated MVs are enriched with ICAM-1 (MVs-ICAM-1^high^) (2.3‐fold ± 0.28) but not HLA-I (1.1‐fold ± 0.06) (Figure 7(c)). Our results show that despite the significant increase in the expression of HLA-I and ICAM-1 in BM-MSCs exposed to an inflammatory environment, the MVs released by these cells are specifically enriched with a cell-cell interaction-mediating molecule, possibly facilitating the interaction of these structures with their target cells.

## 4. Discussion

BM is the main source of MSCs. The study of the biological properties of BM-MSCs generates knowledge that serves as a reference to identify and characterize these cells in other tissues. It is known that the immunoregulatory capacity of MSCs is strongly modulated by the microenvironment. Therefore, intending to induce and increase this function, different laboratories have analyzed the effect of inflammatory cytokines in *in vitro* conditioning protocols, to favor the therapeutic effect of MSCs.

Several studies have indicated the importance of an inflammatory environment with high concentrations of IFN-*γ*for BM-MSCs to carry out efficient immunoregulation [7, 17, 21, 38, 44, 45], and in almost all *in vitro* conditioning protocols, the effect of this cytokine has been analyzed. However, in several cases, IFN-*γ* concentrations that exceed physiological levels are used, even in an inflammatory context, and can affect the morphology [38] and proliferation of MSCs [46–49]. In addition to the above, TNF-*α* has been little studied and has sometimes been used in high concentrations [31, 50].

It has been proposed that IFN-*γ* and TNF-*α* released by activated T lymphocytes are the main cytokines that stimulate the immunoregulatory capacity of MSCs [27, 34, 38]. Based on these antecedents and to manage conditions similar to the physiological context, we analyzed the concentrations of these cytokines in the supernatants of PBMCs activated with PHA for 24, 48, 72, and 96 hours. We detected an increase in the concentration of TNF-*α* after 24 hours, which subsequently decreased over time. While the concentrations of IFN-*γ* increased after 48 hours, these data agree with previous reports [38]. Based on these results, previous studies in our laboratory [13], and reports in which the concentrations of TNF-*α* and IFN-*γ* are detected in nanograms or picograms in the serum or plasma of patients with inflammatory diseases [51–55], we established the concentrations of TNF-*α* and IFN-*γ* to be used in this work.

Several studies have used the increase in MHC expression as a marker of BM-MSC activation, which can predict the efficacy of treatment with inflammatory cytokines and the increase in their immunoregulatory function [7, 12, 38, 56]. Therefore, in the first instance, we analyzed the effect of IFN-*γ* on the expression of HLA-I and ICAM-1. Our data show that all concentrations of IFN-*γ* used increased the percentage of HLA-I^+^ BM-MSCs, which is consistent with previous reports [38, 42, 49]. Interestingly, the minimum concentration of IFN-*γ* (5 ng/mL) at 48 hours increased the percentage of HLA-I^+^ cells slightly higher than the high concentration of IFN-*γ* (90 ng/mL) at 24 and 48 hours. On the other hand, we determined that unstimulated BM-MSCs exhibited low ICAM-1 expression, which is consistent with previous studies [40], and that all concentrations of IFN-*γ* increased the percentage of the cell expressing this molecule in the times analyzed. Our data are supported by other reports, in which treatment with 5-10 ng/mL IFN-*γ* is sufficient to increase ICAM-1 expression [41]. Similar results have been obtained in MSCs from adipose tissue [57] and Wharton's jelly [42]. Besides, the stimulation of MSCs with these concentrations of cytokines induces or increases the expression of immunoregulatory molecules such as IDO, IL-10, COX-2, PD-L1, and TGF-*β* [7, 40, 58, 59]. Taken together, our data show that BM-MSCs can be activated *in vitro* with concentrations of IFN-*γ* closer to those found in the physiological context, which increase the expression of HLA-I and ICAM-1.

TNF-*α* has also been implicated in the regulation of BM-MSC functions; this cytokine is expressed mainly by macrophages, DCs, and T lymphocytes and is capable of modulating the response of cells to other stimuli [29]. It has been suggested that in an inflammatory environment, MSCs are stimulated first with TNF-*α* because it is the first cytokine released by activated T lymphocytes. Therefore, we decided to analyze its effect on the expression of HLA-I and ICAM-1 at early time points. We observed that TNF-*α* did not induce changes in the percentage of HLA-I^+^ cells; however, at 24 and 48 hours, an increase in the expression level was observed. To date, there is only one report of the effect of high concentrations of this cytokine on the increase in the expression of HLA-I [7], but its early effect on the activation of BM-MSCs has not been reported.

Furthermore, this is the first study to report that TNF-*α* is able to increase the early expression of ICAM-1 in BM-MSCs and that exposure to this cytokine beyond 48 hours no longer stimulates the expression of this adhesive molecule. To date, only the increase in ICAM-1 expression on BM-MSCs has been reported after stimulation for 72 hours with high concentrations of TNF-*α* (10 ng/mL), which also induced changes in their morphology [38]. In this sense, our study is relevant because it uses concentrations closer to the physiological context, and in none of our conditions did we observe changes in cell morphology. The decrease in ICAM-1 expression at 72 hours of treatment with TNF-*α* is similar to that reported in lymphatic endothelial cells, which, when exposed to this cytokine for 24 hours, increase ICAM-1 expression, and after 48 hours of stimulation, a decrease is observed [60]. This is important in the induction and regulation of the immune response; it has been shown that the interaction of DCs with the TNF-*α*-stimulated lymphatic endothelium reduces the expression of CD86 in DCs, affecting their ability to induce T lymphocyte proliferation [60]. Similar mechanisms can be used by MSCs because the expression of ICAM-1 strengthens its interaction with immune cells [9, 61, 62]. It has been reported that the overexpression of ICAM-1 in murine MSCs increases their adhesion to DCs, which favors the inhibition of their maturation and differentiation [61]. On the other hand, ICAM-1 participates in the adhesion of MSCs mainly to M1-type macrophages, an event that increases the immunoregulatory potential of MSCs [63]. Besides, blocking this adhesive molecule restores the proliferation of activated T lymphocytes in the presence of BM-MSCs [10, 64] and inhibits the generation of Foxp3^+^ cells [64]. In addition, the administration of BM-MSCs that overexpress ICAM-1 in a murine model of inflammatory bowel disease results in decreased lesions, which is associated with a decrease in the frequency of Th1 and Th17 cells, an increase in regulatory T cells, decreased transcription of IFN-*γ* and Il-17, and increased transcription of Foxp3 [62]. Similar results have been observed in a murine model of graft-versus-host disease [61]. Taken together, this evidence highlights the importance of ICAM-1 in the immunoregulation exerted by BM-MSCs. Knowledge of the stimuli that increase their expression is important to improve *in vitro* conditioning protocols. Therefore, we decided to analyze whether exposure of BM-MSCs to TNF-*α* and IFN-*γ* could further increase the expression of ICAM-1.

The present study shows, for the first time, the synergistic effect of TNF-*α* and IFN-*γ* on the increased expression of ICAM-1 in human BM-MSCs. The above is observed from the first 24 hours of treatment, using low concentrations of IFN-*γ* (5 ng/mL), and is more evident when using high concentrations of this cytokine (90 ng/mL). Similar results have been obtained in murine BM-MSCs [10]. Our results show the relevance of TNF-*α* in the induction and maintenance of the immunoregulatory capacity of MSCs because this property is more efficient when ICAM-1 is highly expressed, as it favors and stabilizes contact with immune cells. This facilitates the effect of secreted and membrane molecules expressed in MSCs. Our observations are supported by previous studies, in which the synergistic effect of TNF-*α* and IFN-*γ* on the stimulation of TGF-*β*, IL-6, and VEGF [65] expression, as well as IDO, PD-L1, and HLA-G [27] expression, was reported. It has even been proposed that exposure of human BM-MSCs to TNF-*α* and IFN-*γ* can cancel the intrinsic variation in each donor relating to the expression of cytokines and chemokines [27] and increase their immunoregulatory capacity [26]. It is important to note that the synergistic effect on the expression of ICAM-1 in BM-MSCs stimulated with high concentrations of IFN-*γ* in combination with TNF-*α* for 48 hours is also accompanied by a higher expression level of HLA-1, which could increase the immunogenicity of these cells [22]. Based on our results, the use of 0.5 ng/mL TNF-*α* and 5 ng/mL IFN-*γ* for 24 hours provided the best stimulation conditions that would allow obtaining the highest percentage of ICAM-1^+^ BM-MSCs.

ICAM-1 is a fundamental molecule in the immunoregulatory function of MSCs because it mediates cell-cell interactions. However, the mechanism by which MSCs can establish direct contact with their target cells in the physiological context is not clear due to their low frequency and graft capacity once administered in patients [66]. Therefore, we analyzed whether BM-MSCs exposed to an inflammatory environment are capable of releasing ICAM-1-containing EVs, which could contribute to their immunoregulatory effect at distant sites.

Importantly, in the literature, there is still confusion in the EV nomenclature [14, 67], which makes it difficult to analyze their biological functions [68, 69]. Currently, EVs are classified mainly based on their cellular origin, size, and shape in exosomes, MVs, and apoptotic bodies. Exosomes are homogeneous vesicles with a size ranging from 40 to 100 nm, are derived from multivesicular bodies (MVBs), and are secreted through a fusion of MVBs with the plasma membrane. On the other hand, MVs are heterogeneous in size, ranging from 100 to 1000 nm, are composed of a lipid bilayer and originate from direct protrusions of the cell membrane that detach from the surface [14, 36]. In the present work, we analyzed the presence of ICAM-1 and HLA-I in MVs released by BM-MSCs.

This study reports, for the first time, that BM-MCSs stimulated with IFN-*γ* release MVs enriched in ICAM-1 (MVs-ICAM-1^high^) but not in HLA-I, which indicates that the enrichment in the cargo of these structures is specific. MVs-ICAM-1^high^ might participate in immunoregulation exerted by MSCs because the presence of ICAM-1 on their surface would allow interaction with target cells and transfer or action from other immunoregulatory molecules. This hypothesis is supported by previous studies that have demonstrated the enrichment of cytokines and chemokines regulating the immune response in MVs released by human BM-MSCs [69, 70]. Furthermore, it has been seen that the MVs released by BM-MSCs activated with IFN-*γ* have a greater capacity to induce the generation of regulatory T cells [71]. On the other hand, the presence of ICAM-1 [72, 73] and the IDO transcript in exosomes released by MSCs stimulated with IFN-*γ* has been identified, as well as the immunoregulatory capacity of these structures [57]. Determining the possible differences in the enrichment of molecules in exosomes and MVs, as well as the impact of this on their physiological functions, is important for the future generation of cell-free therapeutic protocols.

**Figure 8:**
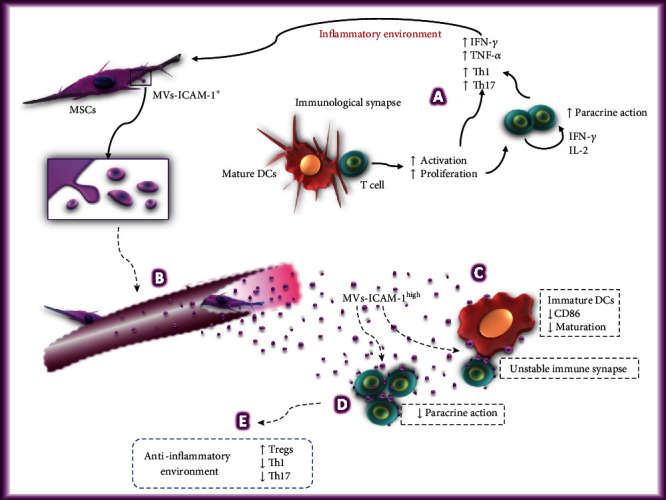
Hypothetical model of the possible mechanisms used by the MVs-ICAM-1^high^ in the immunoregulation of dendritic cells and T lymphocytes. The generation of an inflammatory environment by mature DCs capable of activating T lymphocytes (A) would increase the immunoregulatory capacity of BM-MSCs and stimulate the release of MVs-ICAM-1^high^, which could travel through the bloodstream (B) and contact target cells, such as dendritic cells and T lymphocytes, through ICAM-1. These structures could contribute to the maintenance of DCs in an immature state, unable to adequately stimulate T lymphocytes (C). Likewise, MVs-ICAM-1^high^ could bind to LFA-1 in T lymphocytes, decreasing their ability to form a stable immune synapse, as well as the interaction between them (D). Besides, the direct interaction of MVs-ICAM-1^high^ with DCs or T lymphocytes would also favor the paracrine action of other immunosuppressive molecules transported by these structures. Due to the above, it is possible that MVs replicate the immunosuppression exerted by the whole cell, resulting in the generation of an anti-inflammatory environment in which the proliferation of T lymphocytes and the differentiation of Th1 and Th17 cells decrease, while increasing the differentiation of regulatory T cells (E). Dotted arrows and dotted line text boxes indicate the hypothetical mechanisms that MVs-ICAM-1^high^ could use in the immunoregulation of dendritic cells and T lymphocytes.

Based on our results and previous studies in the literature, we propose a hypothetical model of the possible mechanisms that could involve MVs-ICAM-1^high^ in the immunoregulation exerted by MSCs on DCs and T lymphocytes (Figure 8). It has been demonstrated that the interaction between integrin lymphocyte function-associated antigen-1 (LFA-1) expressed in T lymphocytes and its ICAM-1 ligand expressed in DCs is important for the stability of immune synapses. This has an inflammatory effect because it induces the activation, proliferation, and differentiation of T lymphocytes [74] (Figure 8, A). Exposure of MSCs to this inflammatory environment would increase the release of MVs-ICAM-1^high^, which may travel through the bloodstream (Figure 8, B). It has been shown that ICAM-1 promotes the adhesion of MSCs to DCs, which inhibits the maturation and differentiation of the latter [61]. It is possible that MVs-ICAM-1^high^ can carry out the same effect and contribute to the maintenance of DCs in an immature state, unable to adequately stimulate T lymphocytes (Figure 8, C). Furthermore, MVs-ICAM-1^high^ could bind to LFA-1 on T lymphocytes, reducing their ability to form a stable immunological synapse. On the other hand, it has been reported that T lymphocytes interact with each other through LFA-1 and ICAM-1, both of which are constitutively expressed, although ICAM-1 expression increases in activated T lymphocytes. The said interaction favors the paracrine action of IFN-*γ* and IL-2 [75, 76]. This mechanism could be affected by the binding of MVs-ICAM-1^high^ to LFA-1 in T lymphocytes (Figure 8, D). Besides, the direct interaction of MVs with DCs or T lymphocytes would also favor the paracrine action of other immunosuppressive molecules transported by MVs. In summary, MVs could replicate the immunosuppression exerted by the whole cell, which would result in the generation of an anti-inflammatory environment, in which the proliferation of T lymphocytes and the differentiation of Th1 and Th17 cells decrease, while that increases the differentiation of regulatory T cells (Figure 8, E) [62]. Through *in vitro* and *in vivo* studies, it is necessary to verify whether MV-ICAM-1^+^ could carry out the previously proposed mechanisms.

## 5. Conclusions

In this study, we determined that BM-MSCs can be activated *in vitro* with concentrations of IFN-*γ* and TNF-*α* closer to those found in the physiological context, increasing the expression of HLA-I and ICAM-1. Besides, we observed that TNF-*α* is capable of increasing the early expression of ICAM-1, while its effect on HLA-I occurs later and is less evident. Furthermore, TNF-*α* and IFN-*γ* exert a synergistic effect on the expression of ICAM-1. Finally, we found that BM-MSCs exposed to an inflammatory environment are capable of releasing MVs enriched in ICAM-1 but not in HLA-I. Determining whether these MVs-ICAM-1^high^ can increase the immunoregulation of BM-MSCs at distant sites requires further study. The knowledge generated in this study will contribute to the improvement of *in vitro* conditioning protocols to obtain cells or products with greater capacity for adhesion to immune cells and better immunoregulatory capacity, an indispensable event in tissue regeneration.

## Data Availability

The data on the morphology, immunophenotype, differentiation, cytokine analysis, and isolation and characterization of microvesicles used to support the findings of this study are included within the article.

## Supplementary Materials

Supplementary Materials Supplementary Figure 1: negative controls for adipogenic and osteogenic differentiation protocols. Supplementary Figure 2: high IFN-*γ* concentrations stimulate the higher expression of HLA-I in BM-MSCs.
