# Supplementary material for: Human Bone Marrow Mesenchymal Stem/Stromal Cells Exposed to an Inflammatory Environment Increase the Expression of ICAM-1 and Release Microvesicles Enriched in This Adhesive Molecule: Analysis of the Participation of TNF-α and IFN-γ
Source: J Immunol Res. 2020 Nov 30;2020:8839625. doi: 10.1155/2020/8839625 (PMC7723491; doi:10.1155/2020/8839625)
Supplement: Supplementary Materials — Supplementary Figure 1: negative controls for adipogenic and osteogenic differentiation protocols. Supplementary Figure 2: high IFN-γ concentrations stimulate the higher expression of HLA-I in BM-MSCs. [file 8839625.f1.pdf]

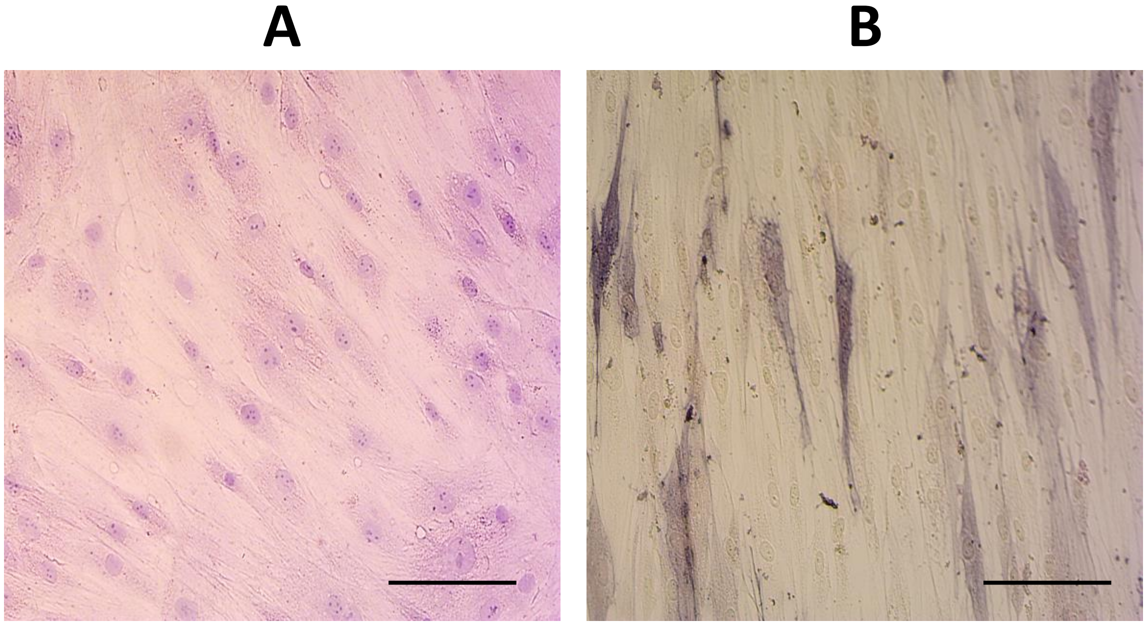

Supplementary Figure 1. Negative controls for adipogenic and osteogenic differentiation protocols. A) Representative image of the negative control of adipogenic differentiation, showing the absence of positivity for oily red. B) Representative image of the negative control of osteogenic differentiation, low positivity for alkaline phosphatase is observed. Scale bar 100µM.

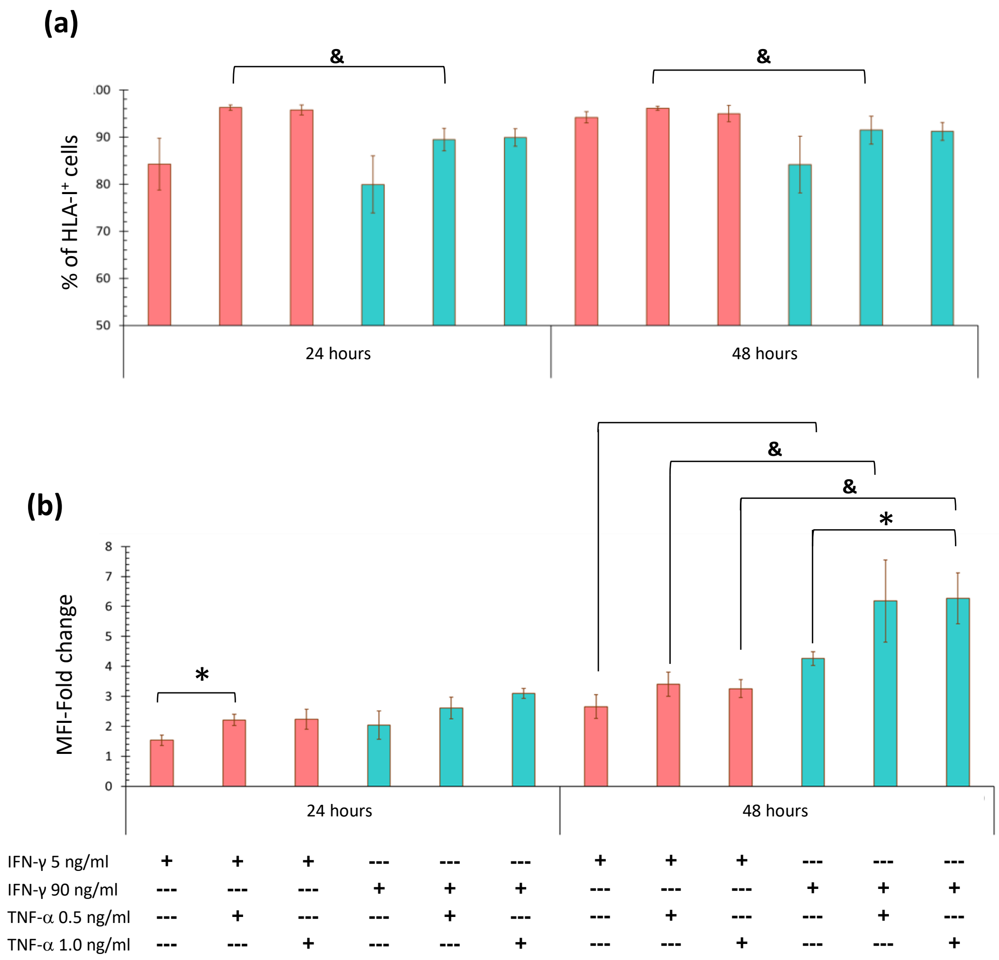

Supplementary Figure 2. High IFN-γ concentrations stimulate the higher expression of HLA-I in BM-MSCs. BM-MSCs were stimulated with 5 and 90 ng/ml IFN-γ alone (Control) or in combination with 0.5 and 1.0 ng/ml TNF-α for 24, and 48 hours. (a) The mean ± SEM of the percentage of HLA-I+ cells; & $p < 0.05$ between 5 ng/ml IFN-γ plus 0.5 TNF-α vs. 90 ng/ml IFN-γ plus 1 ng/ml TNF-α for 24 hours. (b) The mean ± SEM of the fold-change in HLA-I MFI. * $p < 0.05$ with respect to the control. & $p < 0.05$ between 5 vs 90 ng/ml IFN-γ for 48 hours; 5 ng/ml IFN-γ plus 0.5 ng/ml TNF-α vs. 90 ng/ml IFN-γ plus 0.5 ng/ml TNF-α for 48 hours; 5 ng/ml IFN-γ plus 1.0 ng/ml TNF-α vs. 90 ng/ml IFN-γ plus 1.0 ng/ml TNF-α for 48 hours. *n* = 3-7 (independent experiments).
